# Antithymocyte globulin therapy in chronic lung allograft dysfunction

**DOI:** 10.3389/frtra.2025.1607678

**Published:** 2025-07-04

**Authors:** Akhilesh Ajay Padhye, Danielle Guffey, Andres Leon-Pena, Justin Segraves, Ramiro Fernandez, Gabriel Loor, Puneet Garcha, Tianshi David Wu, Gloria Li

**Affiliations:** ^1^ Section of Pulmonary and Critical Care Medicine, Baylor College of Medicine, Houston, TX, United States; ^2^ Institute for Clinical and Translational Research, Baylor College of Medicine, Houston, TX, United States; ^3^ Michael E. DeBakey Department of Surgery, Division of Cardiothoracic Transplantation and Circulatory Support, Baylor College of Medicine, Houston, TX, United States

**Keywords:** chronic lung allograft dysfunction, anti-thymocyte globulin, lung function, FEV1, CLAD, ATG

## Abstract

**Introduction:**

Lung transplantation has seen strides in survival over the past few decades, though long-term survival remains poor. Chronic lung allograft dysfunction (CLAD) is a leading cause of graft failure and mortality beyond the first year. Anti-thymocyte globulin (ATG) is commonly used for treating refractory CLAD, though its efficacy remains uncertain.

**Methods:**

This retrospective study evaluated the impact of ATG on lung function decline and mortality among lung transplant recipients diagnosed with CLAD, defined as a persistent >20% decline in forced expiratory volume (FEV1) from baseline. Patients treated with ATG were compared to those who did not receive ATG, using mixed effects modeling for FEV1 decline and Fine-Gray competing risk modeling for mortality.

**Results:**

Of the 124 patients with CLAD, 55 (44%) received ATG. Administration was not associated with a significant change in FEV1 decline when compared to rate of decline prior to ATG administration [−0.0881 L/year, 95% CI (−0.21, 0.034)] or compared to non-ATG recipients [0.0599 L/year, 95% CI (−0.057, 0.18)]. However, ATG was associated with a lower hazard of all-cause mortality [subhazard ratio 0.66, 95% CI (0.39-1.14)].

**Discussion:**

While ATG improved survival, it did not alter lung function decline, affirming the need for prospective randomized studies.

## Introduction

Lung transplantation has seen significant strides in survival over the past few decades. Despite this, long-term survival remains poor with a median post-transplant survival of 6.0 years (1, 2). One of the leading causes of graft failure and mortality beyond the first year is chronic lung allograft dysfunction (CLAD). This heterogenous disease is characterized by a progressive decline in lung function, encompassing various clinical phenotypes including bronchiolitis obliterans syndrome (BOS), restrictive allograft syndrome (RAS), mixed, and undefined. The pathogenesis of CLAD remains multifactorial, involving alloimmune and autoimmune responses, non-alloimmune injuries, and infections which ultimately lead to loss of graft function (3).

Despite clarity in the definition and diagnosis of CLAD (1), treatment options remain prohibitively limited. Current recommendations include alteration of baseline immunosuppression, augmentation with steroids, azithromycin, or modification of underlying inflammatory risk factors. The benefit from these interventions is modest, and how best to manage patients who progress despite these treatments is unknown (4, 5).

Anti-thymocyte globulin (ATG) is a polyclonal antibody that has a lytic effect on circulating T-lymphocyte cells, leading to a reduction of inflammatory cytokines associated with the alloimmune response in CLAD (3). This theoretical benefit has caused many lung transplant centers to utilize it for salvage therapy for CLAD. Yet, data supporting its use has been largely extrapolated from the renal transplant literature (6).

Prior studies investigating the benefit of ATG in CLAD have focused only on patients who received ATG, examining for efficacy and positive predictive factors. These single-arm studies have yielded mixed results and notably exclude CLAD patients who did not receive ATG, limiting generalizability (7–10). A recent single-center study has demonstrated that ATG was not associated with absolute long-term improvement in lung function in those who received ATG or with survival outcomes, highlighting the need for further research (11).

In this retrospective study of patients with CLAD from a large lung transplant center, we sought to examine whether ATG use was associated with differences in rate of long-term lung function decline or with mortality. Importantly, we compared outcomes with patients with CLAD who did not receive ATG, permitting a novel perspective on ATG's potential impact.

## Materials and methods

### Study population

We performed a retrospective cohort study of lung transplant recipients at Baylor St. Luke's Medical Center from January 2016 to December 2022. Participants were included if they underwent a single, double, or re-transplant during this time and survived to discharge from their initial transplant hospitalization. From this group, we selected participants who developed CLAD (defined based on a persistent >20% decline in FEV1 from the average of the two best post-transplant FEV1 measurements taken at least 3 weeks apart) with progressive decline in lung function. Given the definition of CLAD and its phenotypes were not clarified until 2019 by ISHLT, each patient's CLAD diagnosis was confirmed retrospectively.

### Covariate extraction

Two reviewers (AP, GL) extracted data from the electronic health record using a standardized data collection instrument. In addition to patient demographics, type of transplant, indication for transplant, CLAD phenotype and stage at the time of ATG of administration were collected. Post-transplant related outcomes were collected and included: transplant-related complications [primary graft dysfunction (PGD) at 72 h, presence of deep-vein thrombosis (DVT) or pulmonary embolisms (PE), development of renal failure and explant status for malignancy]. At the date of CLAD diagnosis, maintenance immunosuppression regimen and existence of morbidities was identified, with a special emphasis on the presence of coronary artery disease, chronic kidney disease, gastrointestinal risk factors (GERD, gastroparesis, dysmotility, GI surgery), and presence of DVTs or pulmonary embolism. Finally, any prior diagnosis of acute allograft dysfunction and its associated treatment regimen was noted.

### Treatment with ATG

The primary exposure was whether a participant who developed CLAD received ATG. Because the diagnosis of CLAD is retrospective and based on incorporation of multiple data points, there was heterogeneity in the time from CLAD diagnosis to the receipt of ATG among patients who did receive it; therefore, we classified patients as having received ATG treatment for CLAD if it occurred within three months of their CLAD diagnosis. For patients who received ATG, we recorded treatment-related complications. For patients who did not receive ATG, we identified the reason for not offering this treatment based on chart review.

### Outcomes

The primary outcome was the change in the rate of FEV1 decline on spirometry associated with receipt of ATG. We collected data on frequency of spirometry measurements per person per year prior to, during and after CLAD diagnosis. Because we were interested in examining long-term effects of ATG and due to the heterogeneity in timing of ATG around CLAD diagnosis, we estimated the rate of lung function decline from transplant up to three months before CLAD diagnosis with the rate of decline three months after CLAD diagnosis to the last recorded lung function. We also followed patients from the time of CLAD diagnosis to death due to any cause. With this approach, analyses were conditioned on CLAD diagnosis as the index date, but we were able to avoid potential biases related to the anticipated more frequent spirometry measurements during a CLAD episode.

### Statistical analysis

The rate of lung function change over time was estimated using a linear mixed effects model with random intercept and slope at the participant level with an unstructured covariance structure. Separate indicator variables represented whether measurements occurred before or after the onset of CLAD and whether measurements were derived from participants who received or did not receive ATG. A three-way interaction between these variables with time represented whether the change in the rate of lung function decline associated with CLAD was statistically different between those who did or did not receive ATG. We also calculated linear combinations of these variables from the same model to estimate the mean difference in lung function associated with CLAD and other comparisons. The association of receiving ATG to death was estimated by Fine-Gray competing risk regression, with the competing risk of re-transplant and censored at the time of last follow-up for those who were still alive.

We produced crude and adjusted estimates. Variables for adjustment were chosen based on known predictors of lung function decline or death, CLAD, and likelihood for ATG receipt and thus confound the relationship between ATG and study outcomes. Specifically, we adjusted for age, sex, diagnosis precipitating transplant, best-attained FEV1, number of prior acute rejections, immunosuppressive medication combination, and a comorbidity count of conditions that may impact allograft function or mortality (presence of gastro-esophageal acid reflux disease, coronary artery disease, chronic kidney disease, and diabetes). This adjustment was also applied to the survival analysis. All statistical analyses were performed in Stata 18 (College Station, TX). A two-tailed *p*-value of <0.05 was used to determine statistical significance.

## Results

### Study population

There was a total of 347 unique patients who were transplanted between January 2016 to December 2022. Of these 347 patients, 37 were excluded due to an unknown CLAD status, most commonly because of loss of follow up or death during their transplant hospitalization. Of the 310 remaining patients, 139 were diagnosed with CLAD, 124 of which had a progressive decline in lung function despite initial treatment. These 124 CLAD patients formed our study population. For CLAD phenotype analysis, CLAD was further subclassified as 84 BOS, 21 RAS, 3 mixed, and 16 unknown.

**Figure 1:**
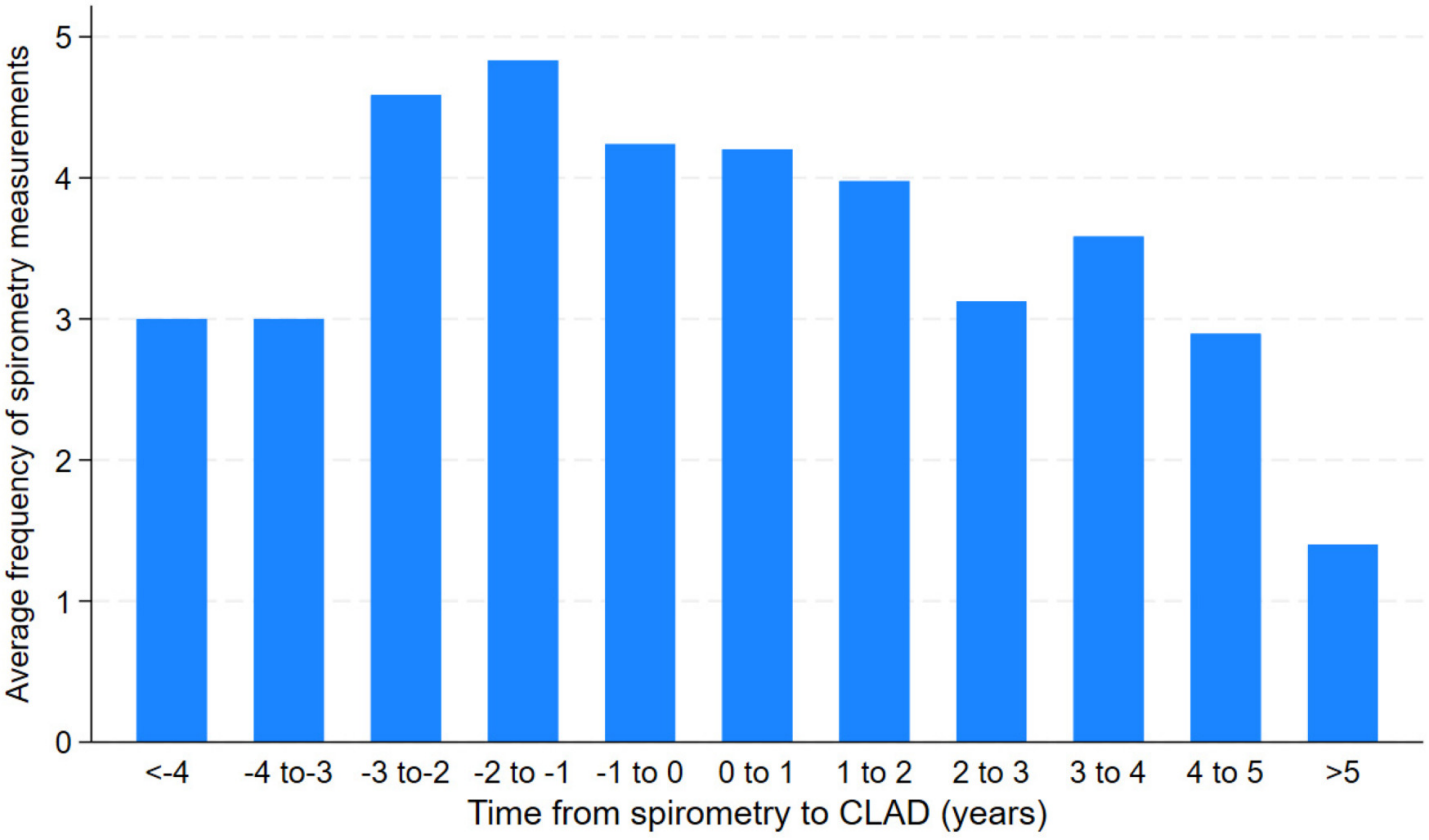
Average frequency of spirometry measurements prior to or after CLAD diagnosis time (years).

Of the 124 included patients, 55 were treated with ATG. Characteristics between those treated and not treated with ATG were similar (Table 1). Specifically, baseline FEV1, CLAD stage, CLAD phenotypes and number of prior acute rejections were similar. However, patients who did not receive ATG had a higher prevalence of malignancy, consistent with malignancy being a relative contraindication to this medication (Table 2). There was also no evidence that spirometry measurements were more frequent outside the three-month period surrounding their CLAD diagnosis (Figure 1).

**Table 1:** Pre-transplant demographics and PGD (primary graft dysfunction) scores of CLAD patients receiving ATG or not receiving ATG.

Demographics	Did not receive ATG (*n* = 69)	Received ATG (*n* = 55)	*p*-value
	Median (IQR)	Median (IQR)	
Age at transplant	59 (44.5,68.0)	58 (34.0,67.0)	0.82
	Number (%)	Number (%)	
Sex			0.71
Male	42 (61)	31 (56)	
Female	27 (39)	24 (44)	
Race			0.86
Asian	1 (1)	1 (2)	
Black of African American	12 (17)	13 (24)	
Hispanic or Latin	15 (22)	11 (20)	
White	41 (59)	30 (55)	
Primary Diagnosis			0.86
Obstructive lung disease	15 (22)	14 (26)	
Cystic fibrosis	15 (22)	9 (16)	
Pulmonary vascular disease	2 (3)	2 (4)	
Restrictive lung disease	37 (54)	30 (55)	
Type of lung transplant			0.58
Single right	9 (13)	6 (11)	
Single left	6 (9)	8 (15)	
Double	54 (78)	41 (75)	
Comorbid conditions			
Diabetes	17 (25)	6 (11)	0.11
Chronic kidney disease (CKD)	13 (19)	5 (9)	0.20
Prior chest surgery	6 (9)	8 (15)	0.39
GERD	32 (46)	32 (58)	0.21
Gastroparesis	2 (3)	3 (6)	0.65
History of GI surgery	9 (13)	9 (16)	0.62
Coronary artery disease	23 (33)	19 (35)	0.85
Index Hospitalization			
PGD at 72 h			0.60
0	6 (9)	5 (9)	
1	26 (38)	17 (31)	
2	25 (36)	18 (33)	
3	12 (17)	15 (27)	

**Table 2:** Baseline FEV1, CLAD staging, active comorbid conditions, prior rejection history, medications, and immunosuppressive regimen during time of CLAD diagnosis.

Follow-Up	Did not receive ATG (*n* = 69)	Received ATG (*n* = 55)	*p*-value
	Mean (SD)	Mean (SD)	
Baseline FVC (L)	2.9 (0.9)	3 (0.8)	0.58
Baseline FEV1 (L)	2.3 (0.8)	2.5 (0.8)	0.37
Transplant to CLAD (years)	2.4 (1.5)	2 (1.2)	0.16
	Number (%)	Number (%)	
CLAD stage at Diagnosis			0.49
1	43 (62)	30 (55)	
2	13 (19)	17 (31)	
3	11 (16)	7 (13)	
4	2 (3)	1 (2)	
CLAD phenotype			0.23
BOS	50 (73)	34 (62)	
RAS	11 (16)	10 (18)	
Mixed	0 (0)	3 (6)	
Undefined	8 (12)	8 (15)	
Number of Prior ACR			0.32
0	37 (54)	23 (42)	
1	24 (35)	19 (35)	
2	6 (9)	10 (18)	
3+	2 (3)	3 (6)	
Number of Prior AMR			0.52
0	53 (77)	46 (84)	
1	13 (19)	6 (11)	
2	3 (4)	3 (6)	
Comorbid conditions at time of CLAD diagnosis			
Coronary artery disease	25 (36)	18 (32)	0.71
Chronic kidney disease	42 (61)	35 (64)	0.85
Diabetes	35 (51)	19 (35)	0.19
GERD	58 (84)	48 (87)	0.80
Gastroparesis	19 (28)	25 (46)	0.04
Esophageal dysmotility	9 (13)	5 (9)	0.57
Diastolic dysfunction	27 (39)	17 (31)	0.35
DVT	8 (12)	6 (11)	1.00
Cancers	12 (18)	1 (2)	0.01
Positive BAL cultures (<1 year)	43 (62)	25 (46)	0.07
Medications at time of CLAD diagnosis			
Azithromycin use	58 (84)	43 (78)	0.49
Montelukast use	10 (15)	7 (13)	1.00
Inhaled steroid use	5 (7)	3 (6)	1.00
Immunosuppresive Medication regimen at time of CLAD diagnosis			0.18
FK + Prednisone	60 (87)	53 (96)	
FK + Sirolimus + Prednisone	3 (4)	2 (4)	
CYA + Prednisone	2 (3)	0 (0)	
Positive BAL cultures (<1 year)	43 (62)	25 (46)	0.07
CYA + MMF + Prednisone	4 (6)	0 (0)	
Graft-related cause of death	30 (71)	26 (84)	0.27

### Association of receipt of ATG with study outcomes

**Figure 2:**
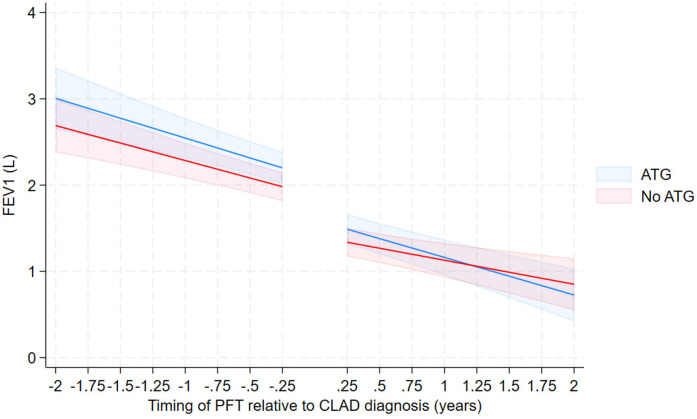
FEV over time from 3-way interaction model—unadjusted with confidence intervals—excluding those with no ATG due to stable lung function.

Among those who received ATG, the rate of FEV1 decline pre and post CLAD diagnosis was −0.35 L/year and −0.44 L/year respectively [difference in rate of −0.0881 L/year, 95% CI (−0.21, 0.034)]. In those who did not receive ATG the rate of FEV1 decline pre and post CLAD diagnosis was −0.33 L/year and −0.27 L/year respectively [difference in rate of −0.0599 L/year, 95% CI (−0.057, 0.18)]. The administration of ATG did not yield a significant difference to the rate of FEV1 decline when comparing the two cohorts [−0.1481 L/year, 95% CI (−0.32, 0.021)] (Table 3; Figure 2). These relationships were not different by CLAD subgroup (not shown).

**Table 3:** Rate of FEV1 decline (L/year), pre and post CLAD diagnosis in patients who did or did not receive ATG.

CLAD therapy	Rate of FEV1 decline before CLAD diagnosis (L/year) (95% CI)	Rate of FEV1 decline after CLAD diagnosis (L/year) (95% CI)	Difference in rate of FEV1 decline after CLAD (L/year) (95% CI)	Difference in difference (L/year) (95% CI)
Received ATG	−0.353 (−0.505, −0.202)	−0.4412 (−0.575, −0.307)	−0.0881 (−0.210, 0.0337)	−0.1481 (−0.317, 0.0207)
Did not receive ATG	−0.3266 (−0.455, −0.198)	−0.2666 (−0.400, −0.133)	0.0599 (−0.0574, 0.177)	

Difference in the rate of decline after ATG administration.

**Figure 3:**
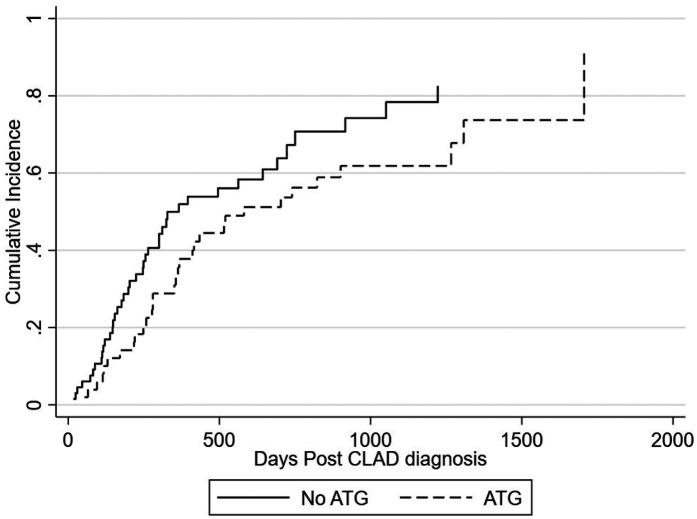
Incidence of mortality after CLAD diagnosis by ATG.

There was a total of 73 deaths. Graft-related cause of death was similar across the two cohorts (Table 2). The 1-year cumulative incidence of mortality post CLAD diagnosis is 43.5% overall, 50.0% for no ATG at diagnosis, and 35.5% for ATG at diagnosis (Figure 3). The adjusted subhazard ratio for ATG received at diagnosis is 0.66 [(0.39–1.14), *p* = 0.134] (Figure 3).

### ATG-Associated treatment complications

In patients treated with ATG, 12 (22%) experienced adverse events during therapy administration: 6 with leukopenia, 2 with serum sickness, 1 with major arrythmia and 3 with various other adverse effects (hypotension, diffuse pain, isolated rash).

## Discussion

In this retrospective cohort study of patients with CLAD cared for at a large volume lung transplant center, we found that treatment with ATG was not associated with a reduction in the rate of lung function decline, both when compared to the rate of decline prior to receipt of ATG and to participants with CLAD who did not receive ATG. These results were consistent irrespective of CLAD subtype. However, those who received ATG had a significantly lower all-cause mortality which was not explained by differences in lung function. Taken together, these heterogeneous results challenge the common clinical practice of using ATG for the treatment of CLAD and highlight the need to investigate the effectiveness of ATG for CLAD in a randomized setting.

Our study is the largest to date examining the role of ATG in CLAD, uniquely utilizing patient datasets that were collected after the establishment of the current definition of CLAD (1). It also marks the first study to directly compare lung function and mortality outcomes in CLAD patients who received ATG vs. those who did not, reflecting real-world clinical practice and offering a broader perspective on CLAD management.

The current ISHLT consensus statement does not list the use of ATG as a treatment option for CLAD, and the data supporting the use of ATG in CLAD is mixed. Dunn and colleagues performed a retrospective review of 63 patients who received ATG for CLAD. While most had a partial response, long-term lung function stability and improved survival was not demonstrated. Izhakian performed a retrospective review of 25 CLAD patients, demonstrating that 68% of patients continued to have deterioration of lung function despite ATG. Irbarnegaray studied 13 CLAD patients who received ATG between 2008 and 2019, finding that over half experienced stable or improved lung function post-treatment, particularly in CLAD Stage 1 or 2. This did not examine long term outcomes beyond 12 months. Finally, Kotecha et al. conducted a larger study involving 67 CLAD patients transplanted over 12 years, showing that approximately 63% responded to ATG. The rate of FEV1 decline improved from 6.5 ml/day to 1.6 ml/day, with a 65% reduction in death or need for retransplant.

Patients who received ATG had lower all-cause mortality compared to those who did not. Because there was no association between receipt of ATG and changes in lung function, which would be expected should ATG be beneficial for arresting CLAD progression, we speculate that this association may be secondary to healthy initiator bias, wherein patients less likely to die due to factors not measured in this study are also more likely to be offered ATG (12). This speculated bias is further evidenced by the noted significantly higher number of malignancies and near significant higher number of positive bronchoalveolar lavage cultures in those who did not receive ATG. An alternative explanation is that ATG may be protective against the sequelae of CLAD in some biologic manner unrelated to lung function; specific mechanisms for such benefit are unclear.

There are several factors that may contribute to the lack of efficacy observed with ATG in CLAD. The pathophysiology of CLAD remains complex and multifactorial, involving not only alloimmune mechanism but also non-alloimmune injuries, autoimmune responses and infections leading to loss of graft function. ATG's primary mechanism of action targets *T*-cell mediated alloimmune response, which represents only a part of the pathophysiology behind CLAD development. This suggests a need for a multipronged approach to halt CLAD progression. The timing and dosing regimen of ATG may also play a role in efficacy. Given that our understanding of ATG is primarily derived from acute rejections or induction therapy literature, the dosing and frequency of use may not be appropriate for the chronic disease that is CLAD. It is possible that repeat administration of ATG may be required to confer a benefit, but this approach poses its own risk of adverse effects or infections.

Our study remains one of the largest to examine the use of ATG in CLAD. However, we acknowledge several limitations. Our study is retrospective and subject to intrinsic limitations of observational studies, including the risk of CLAD misclassification and losses to follow-up. The decision to treat a patient with ATG can plausibly be based on factors unmeasurable in the electronic health record. Additionally, differences in practice and changes to ISHLT CLAD definitions during our study period contributed to heterogeneity in the clinical recognition of CLAD and timing in the receipt of ATG. Finally, our study included patients recruited from a single center, which may limit generalizability. Our center has specific practice patterns utilizing available formulary medications for treatment, compared to other centers which may have access to other treatment modalities such as extracorporeal photopheresis.

In conclusion, this retrospective single-center cohort study of 124 patients with CLAD, receipt of ATG was not associated with a difference in the rate of lung function decline but was associated with a lower all-cause mortality. These findings, taken together with prior mixed results from similar studies, highlight the need for randomized, prospective studies which would mark a significant advancement in our understanding of the role of ATG in CLAD treatment.

## Data Availability

The data analyzed in this study is subject to the following licenses/restrictions: None, they can be generated for review if requested. Requests to access these datasets should be directed to akhilesh.padhye@bcm.edu.
